# A panting behavior-driven assessment framework for summer ventilation quality optimization in layer houses

**DOI:** 10.1016/j.psj.2025.105371

**Published:** 2025-05-28

**Authors:** Zixuan Zhou, Lihua Li, Hao Xue, Yuchen Jia, Yao Yu, Zongkui Xie, Yuhan Gu

**Affiliations:** a College of Mechanical and Electrical Engineering, Hebei Agricultural University, Baoding 071000, China; b Key Laboratory of Broiler/Layer Breeding Facilities Engineering, Ministry of Agriculture and Rural Affairs, Baoding 071000, China; c Hebei Provincial Key Laboratory of Livestock and Poultry Breeding Intelligent Equipment and New Energy Utilization, Baoding 071000, China

**Keywords:** Laying hens, Multimodal, Machine learning, Behavior detection, Ventilation quality assessment

## Abstract

Ventilation quality in summer layer houses is critical for heat stress prevention, production performance, and poultry welfare. Addressing the issue of "qualified environmental parameters but chicken discomfort" caused by traditional methods overlooking spatial heterogeneity and individual differences, a dynamic ventilation quality assessment method based on panting behavior detection in laying hens was proposed. The YOLOv10-BCE panting behavior detection model was developed by embedding the BiFormer module into the backbone network to enhance multi-dimensional feature extraction, compressing neck structure parameters using the C3Ghost module, and integrating Efficient Intersection over Union (EIOU) loss to improve detection accuracy and convergence speed. K-means clustering and linear regression algorithms were employed to establish a quantitative correlation curve between ventilation quality and panting behavior, forming a Normal-Alert-Danger ventilation quality (VQ) classification standard. Experimental results demonstrated that the YOLOv10-BCE model achieved a mean average precision (mAP) of 95.8 % and a detection speed of 0.2 ms, significantly outperforming comparative models such as Faster R-CNN, SSD, and YOLOv9. The ventilation quality correlation model showed high fitting accuracy with an R² value of 0.974. Significant physiological differences (*p* < 0.05) in chickens across VQ grades validated the model's discriminative ability. The method accurately identified latent ventilation anomalies and spatial dead zones in large-scale layer houses. After ventilation strategy optimization, panting prevalence decreased by 65 %, establishing a closed-loop "monitoring-assessment-regulation" dynamic feedback mechanism. This study provides a behavioral-quantitative assessment solution for summer layer house ventilation quality.

## Introduction

Maintaining a suitable poultry house environment is essential for ensuring healthy poultry growth. In modern intensive farming systems, thermal stability is especially emphasized as a critical factor. During summer months, thermal imbalance can induce heat stress in poultry, significantly impairing growth performance, health status, welfare standards, and economic returns (Baumgard and Rhoads, 2013; Wang et al., 2022). Ventilation systems serve as the primary regulatory mechanism for thermal management in poultry houses, making accurate ventilation quality assessment critical for formulating dynamic environmental control strategies.

Current ventilation quality evaluations predominantly rely on sensor-based monitoring systems that track environmental parameters (temperature, humidity, ammonia, and CO₂ concentrations) and equipment operation metrics (fan speed, ventilation rates) (Chen et al., 2014; Rosa et al., 2019; Wen et al., 2022; Yeo et al., 2023; Shin et al., 2024). However, these methods exhibit significant limitations in addressing the dynamic complexity of poultry housing environments. Environmental parameters provide only static or phase-specific snapshots, failing to capture real-time interactions between multiple factors affecting ventilation efficacy. Spatial heterogeneity within poultry houses further complicates assessments, as fixed-point measurements may overlook localized ventilation deficiencies, while high-density sensor deployment faces dual constraints of excessive communication network loads and prohibitive equipment costs. Additionally, traditional evaluation models employ fixed-weight allocation mechanisms that inadequately reflect dynamic physiological responses of poultry, while neglecting breed-specific environmental adaptability variations and regional climatic diversity. This often results in assessment biases where "parameter compliance coexists with flock discomfort."

Laying hen behavior represents an integrative response to environmental stimuli, providing direct insights into their adaptive states under varying conditions (Oladokun and Adewole, 2022). Systematic behavioral monitoring enables precise quantification of hens' physiological responses to ventilation environments, thereby establishing a robust indicator system for ventilation quality assessment that aligns with animal health protection and welfare regulations. Physiologically, modern laying hens—characterized by dense feather coverage, elevated metabolic rates, and absence of sweat glands—primarily regulate body temperature through evaporative cooling via panting behavior (Shakeri et al., 2018; Wasti et al., 2020; Abioja and Abiona, 2021). This thermoregulatory mechanism inadvertently increases localized concentrations of carbon dioxide (CO₂) and carbon monoxide (CO). Inadequate ventilation exacerbates hazardous gas accumulation, creating detrimental environmental feedback loops that compromise flock health. Consequently, panting behavior serves as a critical bioindicator for real-time ventilation quality evaluation in poultry housing systems.

Computer vision has emerged as a transformative tool for non-invasive panting behavior monitoring, offering continuous data acquisition capabilities essential for precision livestock farming (Zu et al., 2023; Yan et al., 2024). Nevertheless, practical implementation in commercial layer houses reveals substantial technical constraints: heterogeneous lighting conditions, inter-animal occlusion patterns, and dynamic background interference collectively degrade algorithmic performance, particularly in high-density rearing environments. Current vision-based detection systems consequently exhibit reduced reliability in operational scenarios requiring real-time decision support.

Addressing the complexity of practical application scenarios in layer houses and the limitations of traditional ventilation assessment methods, this study integrates poultry ethology and environmental engineering to propose a dynamic assessment method for ventilation quality based on panting behavior in layer houses. This method enables continuous, rapid, and precise evaluation of ventilation quality, thereby facilitating the development of rational environmental control strategies.

## Materials and methods

### Experimental design and dataset construction

#### Experimental setup

**Fig. 1:**
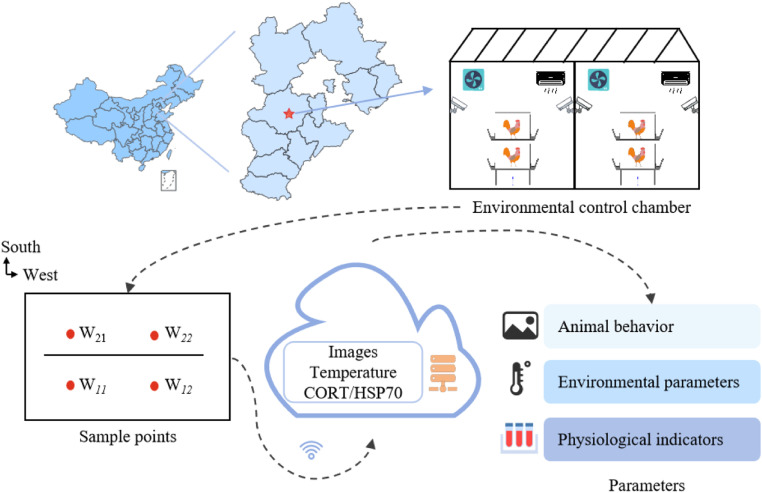
Data acquisition system of AICC.

The study was conducted in AI-controlled climate chambers (AICC), each comprising two independent animal rooms (5.4 *m* × 4.35 *m* × 2.5 m) equipped with a two-tier cage system. A total of 200 laying hens (300 days old) were randomly allocated to two groups (*n* = 100/group): the control group in Room 1 maintained at 23 ± 1°C and 60 ± 10 % RH; The heat-treated group in Room 2 was subjected to intermittent thermal treatments (Table 1) to avoid cumulative effects caused by continuous heat stress and ensure data independence between treatment phases. Specifically, after exposure to acute heat stress, a 24-hour self-regulated recovery period was provided to enable the gradual restoration of physiological homeostasis in chickens before subsequent temperature treatments. This method, which combines segmented heat exposure with recovery periods, effectively prevents the superposition of stress responses and ensures the scientific validity and accuracy of data collection. A tripartite data acquisition system (Fig. 1) synchronously monitored avian behavior, physiological parameters, and environmental metrics.

**Table 1:** Heat treatment schedule.

Day	Heat-treated group	
	Temperature(°C)	Humidity(RH)
1	25 ± 1	60 ± 10 %
2	23 ± 1	60 ± 10 %
3	27 ± 1	60 ± 10 %
4	23 ± 1	60 ± 10 %
5	29 ± 1	60 ± 10 %
6	23 ± 1	60 ± 10 %
7	31 ± 1	60 ± 10 %

**Table 2:** Correlation between temperature and panting behavior.

	Temperature	Panting
Temperature	1 (*P* = 0.000)	0.932 (*P* = 0.012)
Panting	0.932 (*P* = 0.012)	1 (*P* = 0.000)

During the 30-day trial, heat-treated hens underwent daily 6-hour thermal exposure (09:00–15:00) across four phases: Week 1 served as an acclimatization period to mitigate transportation-induced stress; Weeks 2 and 4 implemented graded heat treatments (25°C–31°C); Week 3 reverted to thermoneutral conditions (23°C) to dissipate residual thermal stress. All hens received ad libitum access to feed and water throughout the experimental timeline.

#### Multimodal dataset construction

The study employed a custom-built multi-modal data acquisition system, validated through a 7-day calibration protocol to ensure data accuracy and temporal synchronization across image capture, environmental monitoring, and biochemical sampling. Processed datasets comprised three categories: panting behavior imagery, environmental parameters, and serum biomarkers. Data from Week 2 were used for model development, while Week 4 data served for testing and evaluation.

Image Dataset Construction. Each animal room was equipped with four Hikvision DS-2CD3646FWDA3 cameras (2560 × 1440 pixel resolution at 60 fps), positioned 1.5 m from cages at 45° elevation. Video recordings (09:00–15:00 daily) underwent frame decomposition (1 frame/sec), yielding 11,256 high-quality images after quality screening. LabelImg software annotated panting behavior (open-mouth posture), with data augmentation via MIXUP (scale-chromatic enhancement) and Mosaic (multi-region stitching) techniques to enhance dataset diversity and model generalizability.

Environmental Parameter Monitoring. Four RS-MG111-*−1 air quality transmitters per room (±0.5°C,±3 % RH) recorded temperature and humidity at 1-min intervals. Data were transmitted via 4 G gateways to cloud servers, forming time-synchronized multidimensional environmental matrices.

Serum Biomarker Analysis. Blood samples (5 mL) were collected weekly from 20 randomly selected control hens and post-heat-exposure from 20 treated hens via wing vein puncture. Serum separation involved centrifugation at 3500 rpm for 10 min. Cortisol (CORT) and heat shock protein 70 (HSP70) concentrations were quantified using enzyme-linked immunosorbent assay (ELISA) under standardized laboratory conditions, with blank controls and quality assurance samples ensuring analytical reliability.

### Development and validation of the panting behavior detection model

**Fig. 2:**
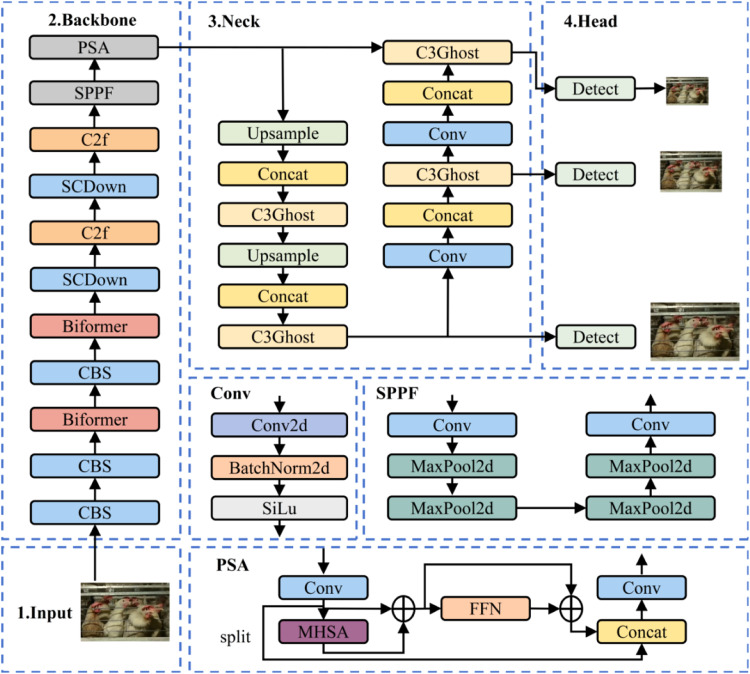
Yolov10-BCE model network architecture.

The proposed YOLOv10-BCE framework comprises three core components: a backbone feature extraction network, a neck feature fusion network, and a prediction module, with the overall architecture depicted in Fig. 2.

The backbone network incorporates BiFormer blocks (Zhu et al., 2023) into its initial two feature extraction layers, enabling multi-scale feature representation through dynamic sparse attention mechanisms. This design enhances the model's capability to capture fine-grained features for small-scale targets across varying resolutions.

Within the neck network, the integration of C3Ghost (Han et al., 2020) modules during upsampling operations facilitates adaptive fusion of multi-scale features. This process eliminates redundant feature channels while preserving critical spatial-semantic information, thereby achieving lightweight feature enhancement through parameter and computational efficiency optimization.

The prediction module employs an Efficient Intersection over Union (EIOU) loss function (Zhang et al., 2022) to refine bounding box regression accuracy. This strategy improves model convergence speed by dynamically adjusting gradient contributions during training while maintaining robust detection performance.

#### Backbone network optimization

**Fig. 3:**
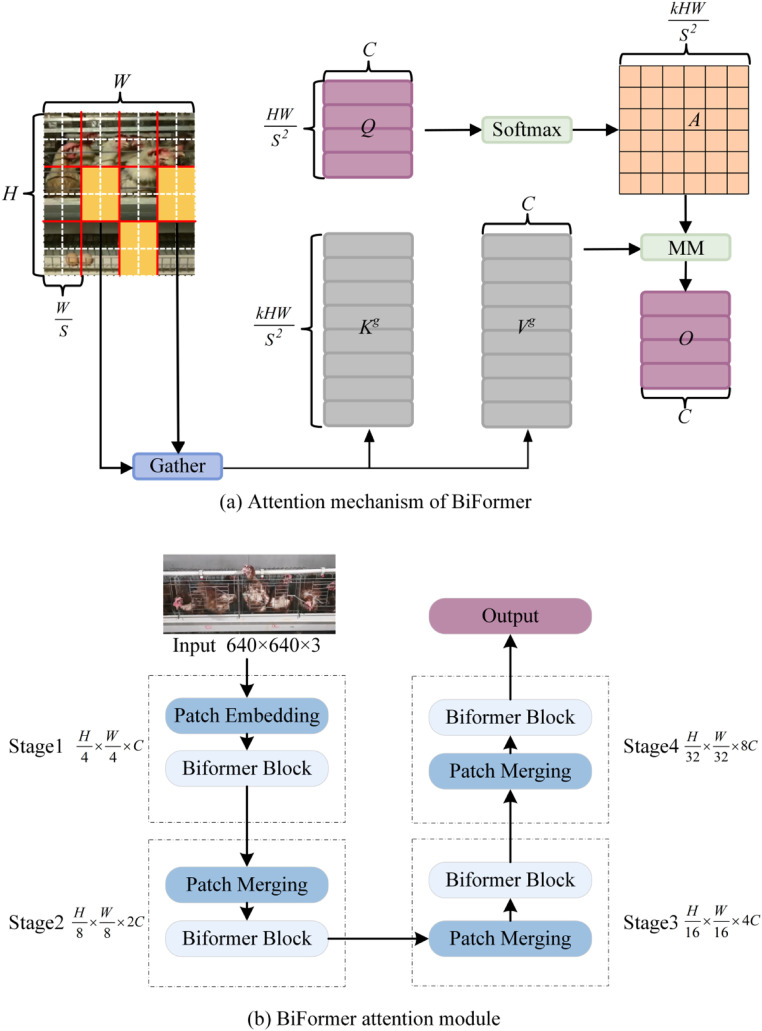
BiFormer module mechanism diagram.

Addressing the challenges of minute size, morphological diversity, and background interference in chicken beak detection, this study proposes an enhanced YOLOv10 detection framework based on BiFormer. As illustrated in Fig. 3(a), we introduce a Dynamic Query-aware Sparse Attention (DQSA) mechanism into the first two feature extraction layers of the network. This module employs a region partitioning strategy to divide the input feature map into *S* × *S* non-overlapping units, generating query (*Q*), key (*K*), and value (*V*) matrices through linear projection (Eq. (1)). The adjacency matrix *A_r_* is constructed via dot-product operations between region-level *Q_r_* and *K_r_* (Eq. (2)), followed by Top-*k* selection to establish a sparse routing index matrix *I_r_* (Eq. (3)). This process effectively aggregates critical regional features to form *K_g_* and *V_g_* tensors (Eq. (4)), which are combined with a local context enhancement term to complete attention computation (Eq. (5)). This adaptive feature enhancement mechanism strengthens target-specific information while suppressing background noise.

The four-stage pyramid architecture, depicted in Fig. 3(b), progressively increases channel dimensions and reduces spatial resolution through overlapping embedding (Stage 1) and block merging operations (Stages 2–4). Each stage contains *N_i_* cascaded BiFormer blocks, whose core components comprise a dynamic weight adjustment mechanism and depth-wise separable convolution (DWConv). The DWConv captures local geometric features through implicit positional encoding, synergizing with attention weight fine-tuning via multilayer perceptrons (MLP). This design significantly improves the model’s discriminative capability for beak edge textures and angular features while reducing parameter counts, effectively resolving the memory-intensive deployment challenges inherent to conventional attention mechanisms.

(1) $$Q=X^{r}W^{q},K=X^{r}W^{k},V=X^{r}W^{v},$$

where *W_q_, W_k_*, and *W_v_* are the projection weights of the query, key, and value, respectively.

(2) $$A^{r}=Q^{r}{\left(K^{r}\right)}^{T}$$

(3) $$I^{r}=\text{topkIndex}\left(A^{r}\right)$$

(4) $$K^{g}=\text{gather}(K,I^{r}),\;\;V^{g}=\text{gather}(V,I^{r})$$

(5) $$O=\text{Attention}\left(Q,K^{g},V^{g}\right)+\text{LCE}\left(V\right)$$

#### Neck network optimization

**Fig. 4:**
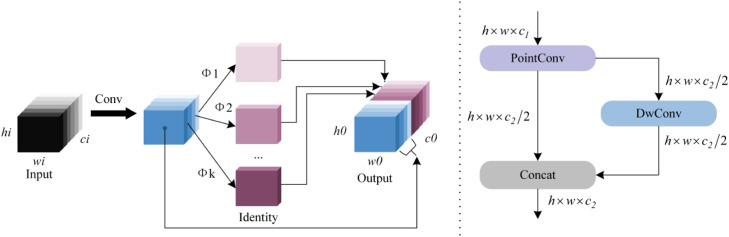
Schematic of the GhostConv module.

To address the increased complexity of the backbone network, this study introduces a C3Ghost module in the neck network for efficient feature fusion. As illustrated in Fig. 4, the module employs a three-stage processing pipeline: First, primary channel features are extracted via convolution-normalization-activation operations. Subsequently, depth-wise separable convolution (DWConv) linearly transforms these primary features to generate ghost channel features. Finally, a feature concatenation strategy merges primary and ghost channels, effectively compressing model parameters and reducing redundancy.

**Fig. 5:**
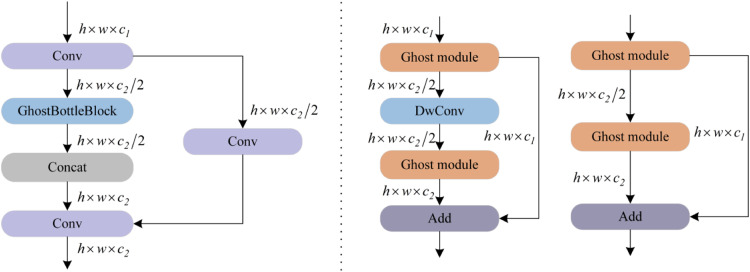
Schematic diagram of the C3Ghost module.

The GhostBottleneck structure shown in Fig. 5 optimizes features through stacked dual-ghost modules. A stride-2 DWConv operation maintains computational efficiency during channel expansion and compression, while skip connections and batch normalization enhance gradient propagation. The integrated GSConv module adopts a channel preservation strategy, replacing traditional convolution with linear transformations to reduce computational costs while preserving feature representation capabilities, thereby offering a practical engineering solution for real-world deployment.

**Fig. 6:**
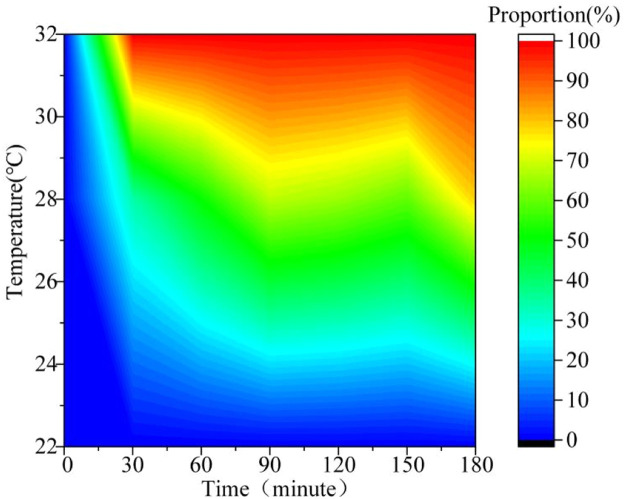
Temporal distribution of panting proportion under different temperatures.

#### Loss function optimization

The precision of bounding box regression directly determines target localization performance. To address the challenges of small-target detection in complex cage environments, this study employs the EIOU loss function for optimized bounding box regression. This function decouples the aspect ratio factor to separately calculate width and height discrepancies between predicted and ground-truth boxes (Eq. (6)). By integrating overlap loss, center distance loss, and dimension loss, it accelerates convergence through minimized discrepancies between ground-truth boxes and anchor points.

(6) $$L_{EIOU}=L_{IOU}+L_{dis}+L_{asp}=1-IOU+\frac{\rho^{2}\left(b,b^{gt}\right)}{c^{2}}+\frac{\rho^{2}\left(w,w^{gt}\right)}{c_{w}^{2}}+\frac{\rho^{2}\left(h,h^{gt}\right)}{c_{h}^{2}}$$

where *b* and *b^gt^* denote the centers of mass of the prediction and truth bounding boxes, respectively, *ρ* denotes the Euclidean distance computed between the two centers of mass, *c* denotes the diagonal distance of the smallest closed region that can contain both the prediction and truth bounding boxes, *w* and *w^gt^* denote the widths of the prediction and ground-truth bounding boxes, respectively, and *h* and *h^gt^* denote the widths of the prediction and truth bounding boxes, respectively.

#### Training and evaluation

In this study, the dataset was randomly divided into training, validation, and test sets in a 7:1:2 ratio. The network training hardware environment consisted of an Intel i7 9700k CPU, an Nvidia RTX3060 GPU, and 64 GB of memory. The network training framework was PyTorch equipped with the parallel computing framework CUDA11.8.This study employed precision (P), recall (R), F1-score (F1), mean average precision (mAP), and model parameter count (Params) to evaluate the performance of the panting behavior detection model, with the formulae presented in Eqs. (7) to (11).

(7) $$Precision=\frac{TP}{TP+FP}$$

(8) $$\text{Re}call=\frac{TP}{TP+FN}$$

(9) $$F1=2\times \frac{\mathrm{Pr}ecision\times Recall}{\mathrm{Pr}ecision+Recall}$$

(10) $$AP=\int_{0}^{1}Precision\times \left(Recall\right)dR$$

(11) $$mAP=\frac{\sum_{i=1}^{N}AP_{i}}{N}$$

True positives (TP) refer to the number of correctly identified positive samples, false negatives (FN) refer to positive samples predicted as negative, and false positives (FP) represent negative samples predicted as positive.

### Development of the ventilation quality assessment model for Layer Houses

#### Quantification of panting behavior for ventilation assessment

Ventilation quality is critical for regulating cage temperature in large-scale poultry production. However, methods evaluating ventilation quality in laying houses based on environmental factors have obvious limitations. They do not fully consider factors such as stocking density and house structure. Poultry behavior results from interactions of multiple environmental factors. These behaviors directly reflect how chickens adapt to their environment. Thus, quantifying behavioral characteristics to build a logical link with ventilation quality can eliminate evaluation biases caused by environmental parameter differences. This study used the proportion of panting behavior (PP)—defined as the ratio of panting chickens to total chickens—as the quantitative behavioral indicator. The experiment was based on an intensive farming model with 8 hens per cage, dividing 200 laying hens into 25 groups in the animal house. Using detection results from the YOLOv10-BCE model for panting behavior, we calculated PP through statistical analysis (Eq. (12)). A total of 33,218 valid data entries were collected during the experiment. Statistical results showed a significant positive correlation between PP and environmental temperature (*P* < 0.05, Table 2). The indicator responded quickly to environmental disturbances and maintained stable data fluctuations within 3 hours (Fig. 6). This assessment method, based on group behavioral statistics, effectively minimizes individual differences. It provides a time-sensitive behavioral basis for evaluating ventilation quality in poultry houses.

(12) $$PP=\frac{\sum_{i}^{n}N_{i}}{nb}$$

**Table 3:** Comparison of recognition results.

Model	P(%)	R(%)	F1(%)	mAP(%)
Yolov10	82.0	80.5	81.2	86.5
Yolov10-BCE	91.5	93.6	92.5	95.8

Where *n* represents the total number of frames, with a value of 300. *b* is the total number of chickens in each group, and *N_i_* is the number of chickens exhibiting panting behavior in the i th frame.

#### Development of the assessment model

**Fig. 7:**
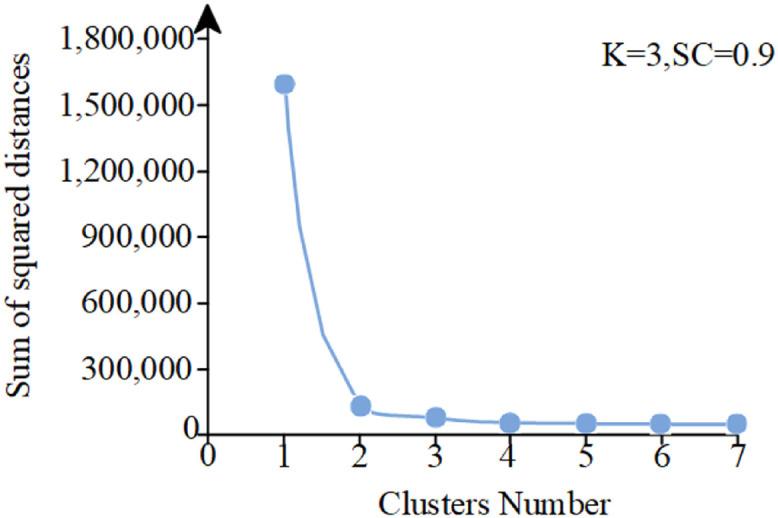
Elbow method analysis for optimal cluster determination.

This study employs K-means clustering to perform unsupervised classification of panting ratio (P) data under diverse environmental conditions. Optimal cluster number determination (*k* = 3, Fig. 7) was achieved through elbow method and silhouette coefficient analysis (*S* = 0.9, Eq. (13)), followed by standardized labeling of P values. A linear regression model with particle swarm optimization (PSO) parameter tuning was subsequently developed to quantify the dynamic response between ambient temperature and panting behavior using P as the predictor variable, establishing a ventilation quality-panting behavior correlation curve (R²=0.974, Eq. (14)).

The ventilation quality (VQ) grading system was established based on regression results: Normal grade: VQ ≤ 1; Alert grade: 1 < VQ ≤ 2; Danger grade: 2 < VQ < 3.

This model quantifies the dynamic relationship between flock behavioral responses and ventilation quality, providing a high-reliability assessment tool for livestock environment monitoring. By establishing measurable correlations between ethological indicators and engineering parameters, it achieves effective translation of behavioral insights into practical ventilation management applications.

(13) $$S=\frac{1}{N}\sum_{i=1}^{N}\frac{b(i)-a(i)}{max\{a(i),b(i)\}}$$

Where *N* is the total number of samples in the dataset. *a*(*i*) represents the average distance from sample *i* to other samples within the same cluster, which reflects the compactness within the cluster. *b*(*i*) denotes the minimum value of the average distances from sample *i* to all samples in other clusters, indicating the degree of separation of sample *i* from other clusters. The closer the silhouette coefficient value is to 1, the better the clustering effect; the closer it is to (−1), the worse the clustering effect.

(14) $$R^{2}=1-\frac{{\sum_{i-1}^{n}\left(y_{i}-{\hat{y}}_{i}\right)}^{2}}{{\sum_{i-1}^{n}\left(y_{i}-{\bar{y}}_{i}\right)}^{2}}$$

Where n is the number of samples, *y_i_* is the i th observed value, $\overset{\wedge }{y_{i}}$is the predicted value of the i th observed value, and$\bar{y_{i}}$ is the mean value of the observed values *y_i_*. The closer the numerical value is to 1, the stronger the model's ability to explain the observed data.

#### Validation of physiological indicators

Serum biochemical parameters serve as critical biomarkers reflecting dynamic physiological state changes. This study selected Cortisol (CORT) and heat shock protein 70 (HSP70) as key indicators. CORT, a primary stress-responsive hormone, directly quantifies environmental stress intensity through its concentration fluctuations (Luijten et al., 2019). HSP70 indirectly evaluates organismal adaptability to adverse conditions by monitoring cellular protein homeostasis (Gouda et al., 2024).

Paired-samples t-test was performed to compare inter-group differences in serum CORT and HSP70 concentrations across ventilation quality (VQ) grades. A statistical significance threshold (*p* < 0.05) was applied to assess mean value discrepancies, testing physiological state variations among VQ classifications. Significant concentration differences between VQ grades would confirm the hierarchical system's capacity to accurately capture physiological differentiation in hens, thereby providing physiological evidence supporting the scientific validity of the ventilation quality assessment model. This approach bridges environmental engineering parameters with molecular biomarkers, establishing a multi-modal validation framework for precision livestock farming.

## Results and discussion

### Performance analysis of the panting behavior detection model

#### Comparison of model feature extraction capabilities

**Fig. 8:**
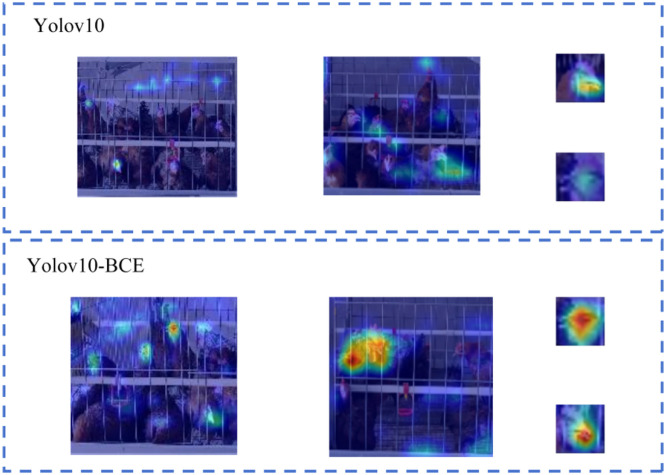
Feature visualization.

This study validated the feature extraction superiority of the YOLOv10-BCE model over YOLOv10 through comparative experiments. Heatmap visualization intuitively revealed data density, distribution patterns, and temporal variations, effectively identifying core regions of interest for model predictions, with color saturation (red intensity in Fig. 8) indicating attention magnitude. Results demonstrated that YOLOv10 exhibited biased heatmap distributions, showing significantly higher global heatmap intensities on cage bars than on chicken head regions, while localized details displayed weak activation on beak and ocular areas. In contrast, YOLOv10-BCE achieved optimized attention allocation, generating peak heatmap intensities on head regions with concentrated activation on beak features, confirming its enhanced capability in precisely localizing critical biological characteristics. This visual evidence substantiates YOLOv10-BCE's architectural improvements in suppressing environmental interference and prioritizing target-specific feature extraction.

Quantitative evaluation results (Table 3) further confirm the superiority of the YOLOv10-BCE model. Compared to YOLOv10, the enhanced architecture demonstrated significant improvements: precision(*P*) increased by 9.5 percentage points to 91.5 %, recall(*R*) improved by 13 percentage points to 93.6 %, F1-score(*F1*) rose by 11.5 percentage points to 81.2 %, and mAP advanced by 9.3 percentage points to 95.8 %. These enhancements stem from the strategic integration of Biformer modules at Layers 1 and 2 of the backbone network, which amplifies feature extraction capability and optimizes small-target detection performance, particularly for avian beak recognition in complex farming environments.

#### Comparison of model lightweighting effects

**Fig. 9:**
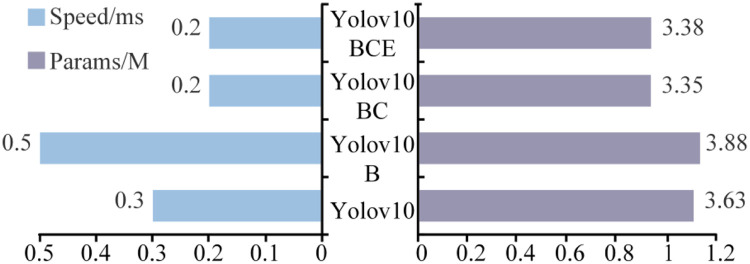
Light weighting test results.

This study systematically evaluated model lightweighting effects through comparative analysis (Fig. 9). The YOLOv10-B model with integrated BiFormer modules exhibited 3.88 million parameters and 0.5 ms inference time, representing a 6.89 % parameter increase and 0.2 ms speed reduction compared to the baseline. In contrast, the YOLOv10-BC model achieved superior lightweighting through C3Ghost modules, reducing parameters by 13.66 % (3.35 million) and improving detection speed by 0.3 ms (0.2 ms) relative to YOLOv10-B. The YOLOv10-BCE variant maintained stable inference speed (0.2 ms) with only 0.89 % parameter increment, demonstrating enhanced parametric efficiency. Experimental results confirmed that C3Ghost modules significantly reduced model complexity through structural optimization while improving computational efficiency, and the EIOU loss function preserved rapid inference capability without parametric overhead, collectively addressing real-time detection requirements.

#### Comparative experiment

**Fig. 10:**
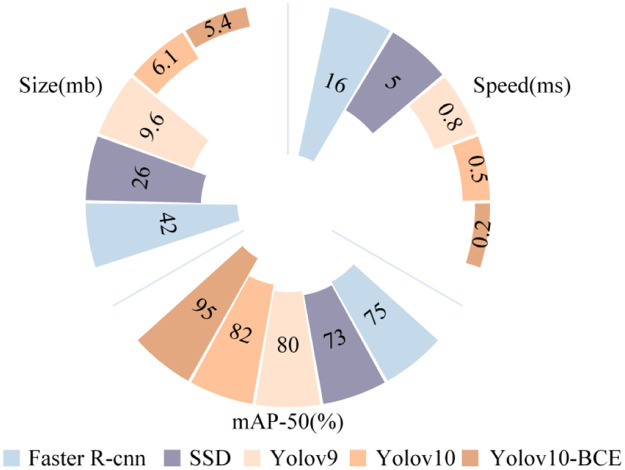
Comparison of detection results of different models.

This study conducted a comprehensive comparative analysis between the proposed YOLOv10-BCE model and mainstream object detection algorithms,Faster R-CNN, SSD, YOLOv9, and YOLOv10 (Liu et al., 2016; Wan and Goudos, 2020; Wang et al., 2024), with results detailed in Fig. 10. Compared to Faster R-CNN and SSD, YOLOv10-BCE demonstrated 20 % and 22 % higher mAP, respectively, while reducing parameters by 36.6 MB and 20.6 MB, and achieving inference speed improvements of 15.8 ms and 4.8 ms. When evaluated against YOLO-series counterparts, YOLOv10-BCE outperformed YOLOv9 and YOLOv10 by 15 % and 13 % in mAP, respectively, with model size reductions of 3.5 MB and 4.2 MB, and detection speed enhancements of 0.5 ms and 0.3 ms. These results substantiate YOLOv10-BCE's comprehensive superiority across critical metrics including detection accuracy, parameter efficiency, and real-time performance, establishing its technical competitiveness in precision-sensitive applications.

**Fig. 11:**
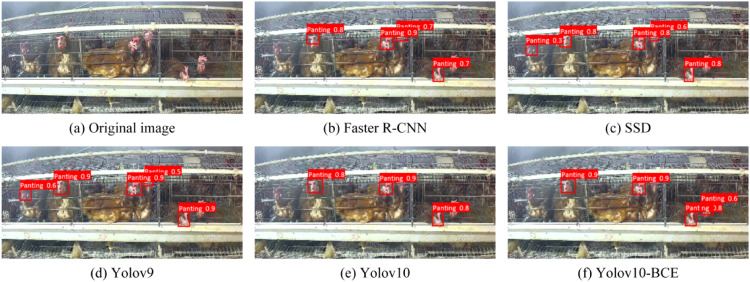
Comparison of detection effects of different models.

Visualized detection outcomes for panting behavior across models are shown in Fig. 10. Faster R-CNN, SSD, and YOLOv9 demonstrated both missed detections and false positives (Fig. 11b-d). While YOLOv10 eliminated false positives, it exhibited detection failures in cage-occluded regions (Fig. 11e). In contrast, YOLOv10-BCE (Fig. 11f) comprehensively detected all panting targets without missed or erroneous identifications. Experimental results confirm YOLOv10-BCE's superior detection efficacy in complex scenarios, with its enhanced robustness demonstrating strong potential for practical deployment.

#### Analysis of model application effects

**Fig. 12:**
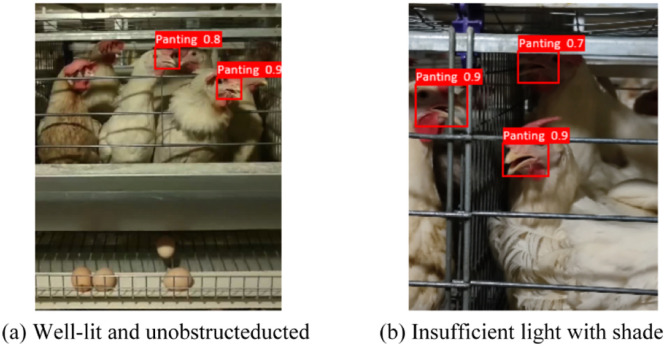
Detection of the effect of closed stacked cages.

The YOLOv10-BCE model demonstrated exceptional panting behavior detection performance in large-scale multi-tier cage layer housess, achieving a mean mAP of 90.2 %. Under scenarios with sufficient illumination and unobstructed visibility (Fig. 12a), strong illumination enhanced the contrast between avian beaks and background, enabling reliable detection accuracy. In complex environments characterized by high-density breeding conditions, multiple occlusions, and insufficient basal lighting (Fig. 12b), YOLOv10-BCE maintained robust detection efficacy. Practical implementation confirmed the model's capability to overcome environmental interference, achieving precise panting behavior identification while exhibiting strong generalization performance. These advancements provide efficient technical support for layer houses ventilation assessment .

### Validation and application of the ventilation quality assessment model

#### Validation of an assessment model

**Fig. 13:**
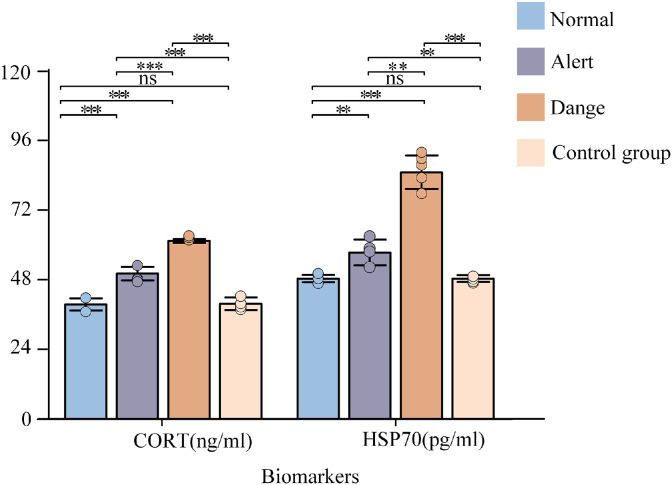
Statistical heterogeneity of biomarkers across ventilation quality levels.

Physiological validation results (Fig. 13) demonstrated no statistically significant differences (*p* > 0.05) in CORT and HSP70 concentrations between the control group and the "Normal" ventilation quality (VQ) tier, while highly significant variations (*p* < 0.05) were observed compared to "Alert" and "Danger" tiers. Inter-tier comparisons within experimental groups revealed pairwise significant differences in CORT concentrations across all three tiers (Normal/Alert/Danger, *p* < 0.05), with HSP70 concentrations showing marked disparities between Normal-Danger (*p* < 0.05) and Alert-Danger tiers (*p* < 0.05).

Distinct physiological differentiations were identified across VQ tiers. Progressive elevation of CORT concentrations with deteriorating ventilation quality reflected intensifying systemic stress, while significant HSP70 upregulation under Danger-tier conditions indicated amplified cellular stress responses. These findings validate the discriminative capacity of the ventilation assessment model in quantifying environmental pressure impacts on poultry physiology. The quantified behavioral monitoring framework provides an actionable solution for real-time ventilation stress alerts in layer houses, with its engineering-compatible evaluation model demonstrating practical potential to advance precision environmental management in intensive poultry production systems.

#### Application in commercial-scale layer houses

Through standardized environmental controls and physiological response validation, this study established a panting behavior detection model and ventilation quality assessment method. We implemented the proposed method to evaluate ventilation systems in commercial layer houses. During an eight-day experimental period, spatio-temporal distribution patterns of both environmental parameter-based method and behavioral assessment method were recorded. Finally, a comparative experiment was conducted to systematically evaluate the applicability and robustness of the behavioral assessment method in layer houses.

**Fig. 14:**
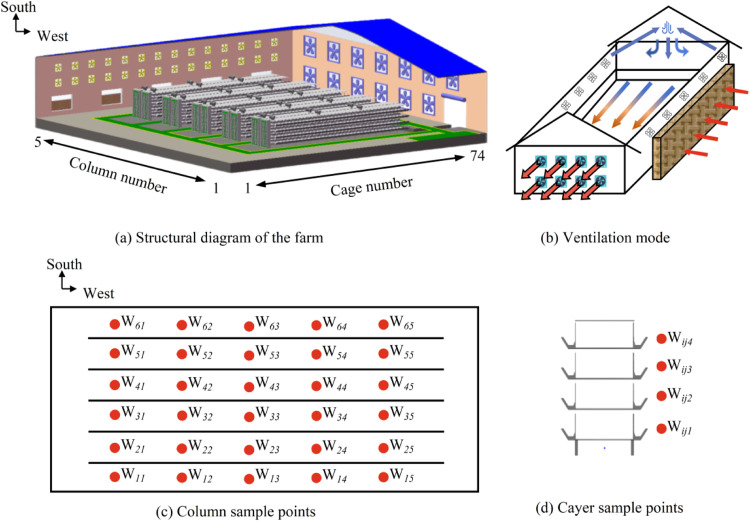
Schematic diagram of sampling points in the layer house.

The experimental layer house (100 *m* × 15 *m* × 40 m) located in Shahe City, Xingtai, adopted a five-row four-tier stacked cage system with a total capacity of 41,440 birds (8 birds per cage, Fig. 14a). It also integrated a combined positive-negative pressure ventilation system (Fig. 14b). Environmental parameters and video data were synchronously collected via a custom-built monitoring system. Following the principle of equidistant sample point placement, five sample points were arranged horizontally in each column (Fig. 14c). In the vertical direction, four sample points were established (Fig. 14d). This resulted in a total of 120 monitoring points. The sample points simultaneously collected temperature data and video on a daily basis from 08:00 to 18:00.

##### Ventilation time period assessment

**Fig. 15:**
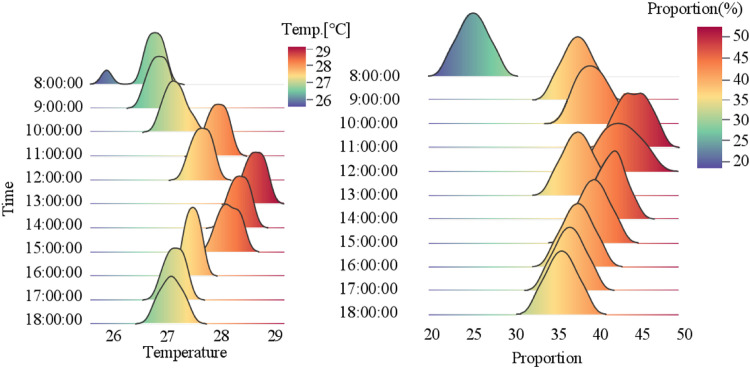
Temporal variation in temperature and panting proportion within the layer house.

This study implemented continuous monitoring of layer houses temperatures and panting behaviors, employing ridge plot visualization to capture daytime dynamics over 8 consecutive days (Fig. 15). Monitoring data revealed cyclical temperature fluctuations peaking between 12:00–14:00 (29°C) followed by gradual cooling, while panting behavior proportions mirrored thermal trends. Analytical results identified that temperature threshold was exceeded in only 3 midday periods, yet significant panting behavior was detected in 10 of 11 daytime monitoring intervals.

**Fig. 16:**
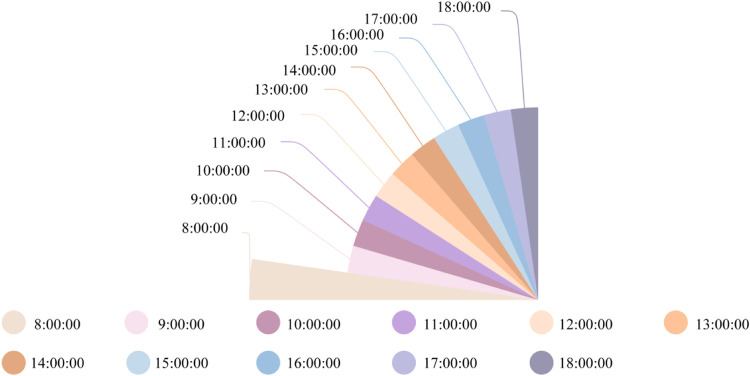
Time-resolved ventilation quality diagnostics in the layer house.

The proposed ventilation assessment framework was systematically applied to evaluate daytime environmental conditions. Polar plot visualization (Fig. 16) quantified ventilation tiers across daytime periods over 8 days, with radial axis lengths representing performance metrics. Data indicated Normal-tier ventilation solely at 08:00, while Alert-tier conditions persisted throughout other daytime periods.

Comparative analysis demonstrated that the ventilation anomaly detection method developed in this study achieved high precision. Specifically, the method was shown to significantly enhance the capability of detecting the effectiveness of ventilation regulation in laying houses.

##### Spatial ventilation assessment in layer houses

**Fig. 17:**
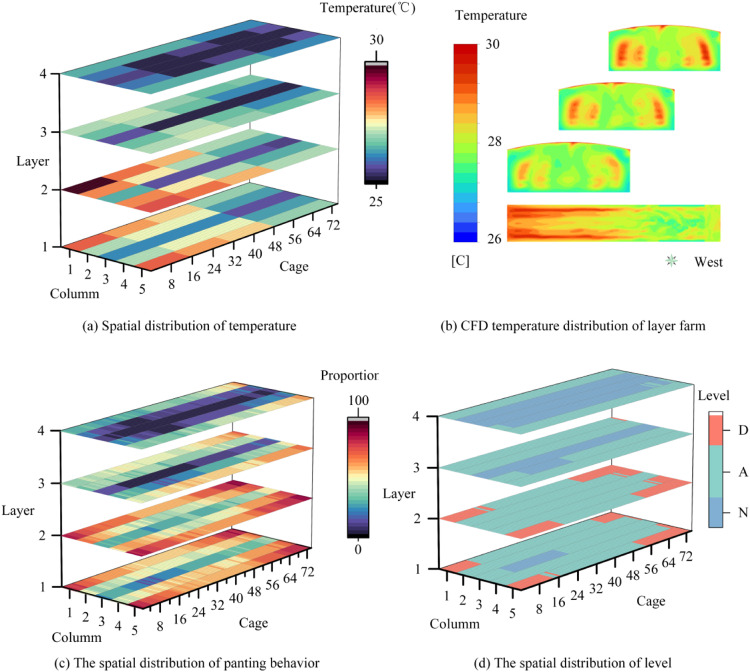
Spatial distribution of temperature gradients, panting proportion, and ventilation assessment grades in the layer house.

This study implemented the proposed assessment methodology to evaluate spatial ventilation performance in poultry facilities. Fig. 17 illustrates the three-dimensional distributions of temperature, panting prevalence, and ventilation tiers, with spatial coordinates defined by X-axis (Column), Y-axis (Cage), and Z-axis (Layer).

Thermal mapping (Fig. 17a-b) revealed facility-wide temperatures within 25-30°C. Maximum temperatures (27-30°C) occurred at Layers 1-2 of Columns 1/5 and Layer 2 of Columns 2/4, while Layers 3-4 maintained the lowest range (25-26°C). CFD-simulated thermal fields (Fig. 17b) confirmed east-high-west-low and edge-high-center-low gradients through cross-sectional and planar visualizations.

**Fig. 18:**
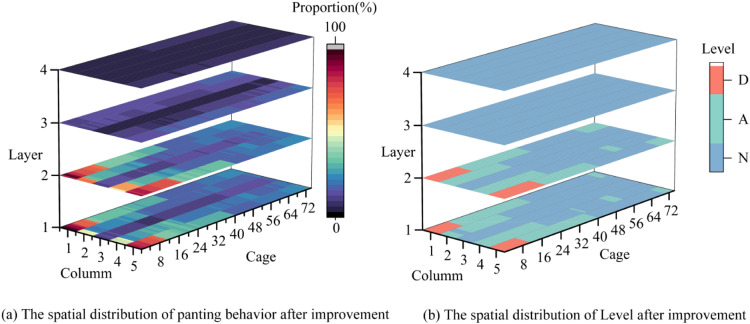
Post-improvement spatial mapping of panting proportion and ventilation assessment grades in the layer house.

Fig. 17c-d present the spatial distribution of panting behavior within the livestock farm. Fig. 18c demonstrates elevated panting prevalence, with red zones predominantly distributed across Columns 1 and 5. At Layer 2, these high-stress zones extended to terminal sections of Columns 2-3, maintaining equivalent panting levels. In contrast, blue and purple zones at Layers 4-5 indicated moderate panting activity. Fig. 17(d) shows the evaluation distribution results of each cage in the layer houses. Ventilation tier mapping in Fig. 17(d) reveals critical environmental disparities. Alert-tier conditions dominated the majority of cages, with Danger-tier clusters concentrated at corner positions in Layers 1-2. Normal-tier zones were exclusively identified in central-column cages of Layers 3-4.

Notably, temperature-appropriate conditions (25-28°C) coexisted with localized panting hotspots. Mechanistic analysis attributed this paradox to ventilation imbalances: inadequate positive-pressure fan capacity allowed cold airflow to escape before reaching edge/basal regions, while excessive negative-pressure extraction exacerbated eastern/basal dead zones. Post-optimization adjustments (disabling upper negative-pressure fans) reduced overall panting by 65 %, with notable improvements in central/lower zones (Fig. 18a-b). Persistent Danger-tier in eastern basal Layers 1-2 highlighted residual airflow deficiencies.

This panting-based methodology successfully overcame spatial heterogeneity limitations inherent to conventional temperature monitoring, providing actionable insights for precision ventilation management in industrial poultry operations.

## Conclusions

This study introduces a panting behavior-based methodology for summer ventilation quality assessment in layer houses. To address illumination heterogeneity and occlusion challenges in complex farming environments, an enhanced YOLOv10 detection model (YOLOv10-BCE) was developed to enable real-time panting recognition. The BiFormer module was embedded in the Backbone network to optimize feature extraction capabilities. The lightweight C3Ghost module was utilized to reconstruct the FPN, thereby reducing computational complexity. The EIoU loss function was used to improve both the regression accuracy and convergence speed of the model. Using panting proportion as the proxy variable, a Normal-Alert-Danger ventilation quality grading standard was established via machine learning algorithms. Ablation studies, comparative experiments, statistical analyses, and field applications collectively validated the method's significant advantages in ventilation assessment.

(1) The YOLOv10-BCE model demonstrated superior panting detection performance, achieving a 9.5 % increase in mAP (final 95.8 %) with 13.66 % parameter reduction while maintaining an efficient detection speed of 0.2 ms/frame. In multi-tier cage systems, the model attained 90.2 % mAP, showcasing enhanced robustness and generalizability that provide high-efficiency technical support for layer houses ventilation assessment.
(2) The panting-based ventilation quality assessment method exhibited high accuracy and reliability, with the three-tier Normal/Alert/Danger model achieving a coefficient of determination R²=0.974. Statistically significant physiological differentiations (*p* < 0.05) between ventilation tiers validated the model's discriminative capacity. Large-scale layer house validation demonstrated its capability to precisely identify latent ventilation anomalies and spatial dead zones, establishing a closed-loop "monitoring-assessment-regulation" dynamic feedback mechanism through ventilation strategy optimization. These findings provide a novel behavior-environment coupling monitoring approach for livestock environmental control.

## Declaration of competing interest

The authors declare that they have no known competing financial interests or personal relationships that could have appeared to influence the work reported in this paper.
